# Enhanced End-Contacts by Helium Ion Bombardment to Improve Graphene-Metal Contacts

**DOI:** 10.3390/nano6090158

**Published:** 2016-08-26

**Authors:** Kunpeng Jia, Yajuan Su, Jun Zhan, Kashif Shahzad, Huilong Zhu, Chao Zhao, Jun Luo

**Affiliations:** 1 Key Laboratory of Microelectronic Devices & Integrated Technology, Institute of Microelectronics of Chinese Academy of Sciences, 3 Bei-Tu-Cheng West Road, Chaoyang District, Beijing 100029, China; jiakunpeng@ime.ac.cn (K.J.); zhanjun@ime.ac.cn (J.Z.); kashif@ime.ac.cn (K.S.); zhuhuilong@ime.ac.cn (H.Z.); zhaochao@ime.ac.cn (C.Z.); 2 University of Chinese Academy of Sciences, Beijing 100049, China

**Keywords:** graphene, contact resistance, helium ion bombardment, defects, end-contact

## Abstract

Low contact resistance between graphene and metals is of paramount importance to fabricate high performance graphene-based devices. In this paper, the impact of both defects induced by helium ion (He^+^) bombardment and annealing on the contact resistance between graphene and various metals (Ag, Pd, and Pt) were systematically explored. It is found that the contact resistances between all metals and graphene are remarkably reduced after annealing, indicating that not only chemically adsorbed metal (Pd) but also physically adsorbed metals (Ag and Pt) readily form end-contacts at intrinsic defect locations in graphene. In order to further improve the contact properties between Ag, Pd, and Pt metals and graphene, a novel method in which self-aligned He^+^ bombardment to induce exotic defects in graphene and subsequent thermal annealing to form end-contacts was proposed. By using this method, the contact resistance is reduced significantly by 15.1% and 40.1% for Ag/graphene and Pd/graphene contacts with He^+^ bombardment compared to their counterparts without He^+^ bombardment. For the Pt/graphene contact, the contact resistance is, however, not reduced as anticipated with He^+^ bombardment and this might be ascribed to either inappropriate He^+^ bombardment dose, or inapplicable method of He^+^ bombardment in reducing contact resistance for Pt/graphene contact. The joint efforts of as-formed end-contacts and excess created defects in graphene are discussed as the cause responsible for the reduction of contact resistance.

## 1. Introduction

In the past decade, graphene has attracted worldwide attention as a miracle material with unique electrical and physical properties [1,2,3]. Graphene holds great promise in a range of device applications, such as field effect transistors, photo-detectors, radio frequency (RF) devices, and spintronics [4,5,6,7,8]. For all these application scenarios, the large specific contact resistance (R_c_) between graphene and metals is one of grand challenges which hinders the progressive improvement on device performance [9,10,11,12,13,14,15,16,17,18]. In order to access the intrinsic excellent electrical properties of graphene, the R_c_ between graphene and metals as low as possible is desired. There has been plentiful studies on the properties of graphene-metal (G-M) contact [11,16,19,20,21] and methods to reduce G-M contact resistance. Choi et al. [22] and Robinson et al. [23] proposed a plasma treatment to make the graphene surface hydrophilic, thus enhancing the bonding between graphene and metals. By using this method Robinson got as low contact resistance as 10^−7^ Ω·cm^2^. Similar to this plasma treatment, ultraviolet/ozone (UVO) was used to clean the graphene surface after lithography which reduced contact resistance to 7 × 10^−7^ Ω·cm^2^ and 568 Ω·μm respectively [24,25]. Nevertheless, due to the difficulty in tuning the processing parameters properly when applying these methods, the excellent uniformity in reduced R_c_ over a wide range cannot be easily realized, or even the R_c_ will be degraded under some circumstances. Alternatively, the graphene/metals end-contacts, initially disclosed in a theoretical work [26], is claimed to possess great potential in improving R_c_ dramatically. The utilization of end-contacts in reducing R_c_ is also confirmed by experimental efforts [20,27,28]. In those works, notably reduced R_c_ were demonstrated by introducing holes (~2.2 × 10^−9^ Ω·cm^2^) [20], etched zigzag edges (100 Ω·μm) [27], or line cuts (125 Ω·μm) [28] in the contact area of graphene to form end-contacts with metals. It is worth noting that although R_c_ can be reduced using end-contacts, the approach to introducing various patterns in the contact area of graphene is too complicated to be implemented in practical applications, since in these cases the lithography and subsequent dry etching is usually needed in order to shape the holes, zigzag edges, or line cuts [20,27,28]. Moreover, this additional lithography for introducing patterns in the contact area of graphene may incur the problem of polymer residues on graphene, which would possibly degrade R_c_. In this regard, a simple while efficient method featuring the end-contacts concept to reduce R_c_ is of great importance and interest. In this work, a novel method to form end-contacts between graphene and various metals (Ag, Pd, and Pt) is explored. In this method, defects are introduced to graphene by light He^+^ bombardment and thereafter end-contacts are formed between graphene and metals. Since the thermal annealing treatment is a common yet effective way to reduce R_c_ [29,30], the effect of annealing on R_c_ with and without He^+^ bombardment is also investigated in this work.

## 2. Experiment

**Figure 1:**
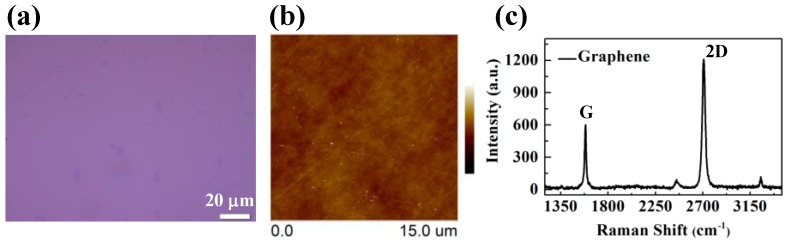
(**a**) Optical and (**b**) Atomic force microscopy (AFM) images of chemical vapor deposition (CVD)-grown graphene that is transferred onto SiO_2_/Si substrate; (**c**) showing the Raman spectrum of the as-transferred graphene. The color scale of AFM image is −20–20 nm.

Thermal chemical vapor deposition (CVD) grown graphene [31,32] was transferred onto a heavily p-type doped silicon wafer with 100 nm thermal oxide [33]. The optical microscopy (OM) and atomic force microscopy (AFM) were employed to inspect the morphology of transferred graphene, as shown in Figure 1a,b. The results display that the graphene film is continuous with a large single layer ratio. To further characterize the quality of the graphene, Raman scattering spectroscopy was implemented and the Raman spectrum is shown in Figure 1c. The Raman scattering measurement was performed in air using a 50× objective and the excitation laser energy was 2.41 eV (514 nm). Low laser power was used to avoid the sample damage caused by heating. The G peak and 2D peak appearing at ~1585 and ~2700 cm^−1^ respectively are observed and the ratio of 2D/G peaks is higher than 2 indicating that the transferred graphene is single layer.

**Figure 2:**
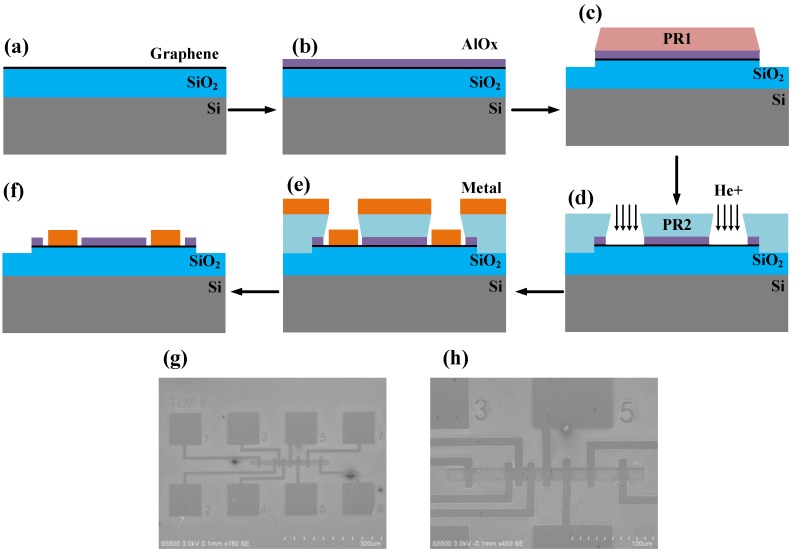
Schematics of the process showing the fabrication flow of transferring line method (TLM) test structures. (**a**) CVD-grown graphene is transferred onto SiO_2_/Si substrate; (**b**) The AlO*_x_* isolation layer is deposited on graphene by Atomic Layer Deposition (ALD); (**c**) The definition of graphene diffusion strip by the first lithography and plasma etching; (**d**) The opening of contact windows by a second lithography and wet etching (Afterwards, optional He^+^ bombardment); (**e**) Metals are deposited by e-beam evaporation; (**f**) Lift-off process to remove unwanted photoresist (PR) and metals; (**g**,**h**) showing the scanning electron microscope (SEM) images of as-fabricated TLM test structures. The gap between two G-M contacts varies from 3 to 40 μm.

Transferring line method (TLM) is used to extract the contact resistance between graphene and metals. The fabrication of TLM test structures is schematically shown in Figure 2a–f. Note that conventionally in the fabrication of graphene-based devices, photoresist (PR) is spin-coated directly on graphene and thereafter the contact window is opened by lithography. In such a case, PR residues usually occur which cannot be removed effectively. PR residues not only influence the properties of graphene but also impede the good contact between graphene and metals. In order to eliminate the adverse effect of PR residues on the contact properties, a ~12-nm-thick aluminum oxide (AlO*_x_*) isolation layer is deposited between PR and graphene as a hardmask by Atomic Layer Deposition (ALD) [34] (cf. Figure 2a,b). The graphene diffusion strips were protected by patterned positive PR followed by Ar plasma etching to remove un-protected AlO*_x_* and underneath graphene (cf. Figure 2c). After the definition of a graphene diffusion strip, another lithography using negative PR was used to define the contact windows followed by wet etching using dilute solution of H_3_PO_4_ (1:3) at 40 °C to remove the unwanted AlO*_x_* layer in these contact windows (cf. Figure 2d). Afterwards, the samples were categorized into two sets. For the first set without He^+^ bombardment, metals (X/Au = 40/10 nm, X = Ag, Pd or Pt) were deposited directly on graphene by e-beam evaporation (cf. Figure 2e). For another set, the contact windows were shot by energetic He^+^ and then the same metals were deposited by e-beam evaporation (cf. Figure 2d,e). The fabrication of TLM test structures was finished by a lift-off process to remove unwanted PR and metals (cf. Figure 2f). For both sets, the TLM test structures were electrically characterized before and after a thermal annealing. The thermal annealing process was implemented in a tube furnace at 420 °C/30 min under a low pressure of 40 Pa. During the whole annealing process, 50 sccm argon (Ar) gas was steadily pumped into the tube furnace. Scanning electron microscope (SEM) images of as-fabricated TLM test structures are shown in Figure 2g,h. The width of defined graphene diffusion strips is 15 μm and the gap between two G-M contacts varies from 3 to 40 μm. The characterizations of specific contact resistance for different G-M contacts were performed using a Keithley 4200 semiconductor parameter analyzer (IMECAS, Beijing, China).

## 3. Results

### 3.1. Effect of Annealing on the G-M Contact Properties

**Figure 3:**
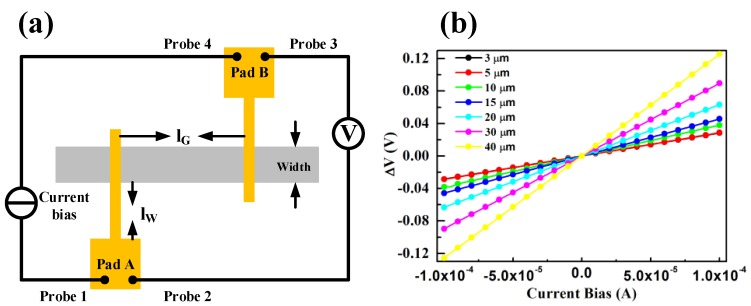
(**a**) Four probe configurations and (**b**) measured current-potential drop characteristics of typical G-M contact with different l_G_ in TLM test structures.

For the extraction of G-M contact resistance, four probe configuration illustrated in Figure 3a is employed in TLM test structures. The current flows from probe 1 to probe 4 and the voltage drop is measured between probe 2 and probe 3 at the same two landing pads. The ideal linear relationship between voltage drop and input current shown in Figure 3b demonstrates good ohmic contacts between graphene and metals. It is well known that, for the TLM test structures, the measured total resistance (R_T_) consists of graphene sheet resistance (R_S_), G-M contact resistance (R_GM_), metal wire resistance (R_W_), and probe-pad contact resistance (R_PP_) as seen in Equation (1).

(1) $$R_{T}=R_{S}\left(l_{G}\right)+2R_{\text{GM}}+R_{W}\left(l_{W}\right)+2R_{\text{PP}}$$

**Figure 4:**
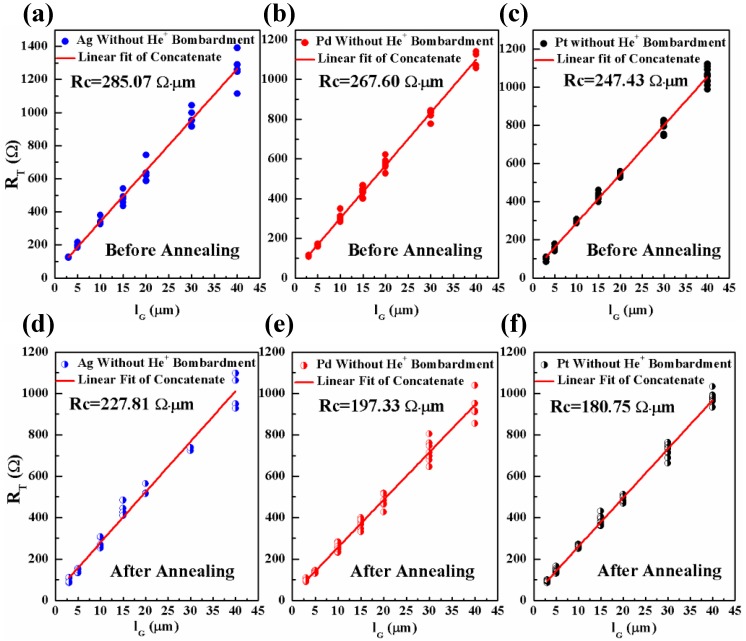
The linear plots of R_T_ vs. l_G_ for the contacts between Ag, Pd, Pt and graphene without He^+^ bombardment before annealing (**a**–**c**) and after annealing (**d**–**f**). For each l_G_, 10 data points are used and the liner fitting is performed using concatenate fit method. Extracted R_c_ value is shown in each corresponding plot.

Among them, R_S_ relates to the length of graphene diffusion strip (l_G_) between two G-M contacts and the value of R_W_ depends on the length of metal wires between pads and G-M contact windows (l_W_). Benefitting from the four point probe configuration in TLM method, R_PP_ can be ignored. The slope in Figure 3b yields R_T_ which is the sum of R_S_, 2R_GM_, and R_W_. Normally, R_w_ is small which can be omitted in Equation (1) if metal wires are thick and/or short. However, since in this work the deposited metal wires are thin i.e., 50-nm-thick and long, R_w_ cannot be omitted which is also a disturbing factor for the linear plotting of R_T_ vs. l_G_. In order to get a perfect linear plotting, it is a must to subtract R_w_ from R_T_. The sheet resistances of metal wires are extracted to be 0.96, 3.72 and 4 Ω/□ for Ag/Au, Pd/Au and Pt/Au, respectively. As a result, the values of R_w_ can be calculated according to l_W_ and the width of metal wires and be subtracted from R_T_. After the R_w_ subtraction, perfect linear relationships between R_T_ and l_G_ for all G-M contacts are accomplished as shown in Figure 4.

For the contacts between Ag, Pd, Pt, and graphene without He^+^ bombardment, the linear plots of R_T_ vs. l_G_ before annealing (a–c) and after annealing (d–f) are shown in Figure 4. For each l_G_, 10 data points are used and the linear fitting is performed using concatenate fit method. The perfect linear fittings for all G-M contacts are evident demonstrating the validity of employed method to extract R_c_ in this work. The interception of the fitted red line with R_T_ (*Y*-axis) yields the value of 2R_GM_. Since the width of the graphene diffusion strip is 15 μm, R_c_ is therefore calculated by R_GM_ × 15 μm which is also offered in each corresponding plot. For all three G-M contacts, it is clearly observed that R_c_ values are reduced significantly after annealing which confirms the effectiveness of the annealing in improving G-M contacts [30].

**Figure 5:**
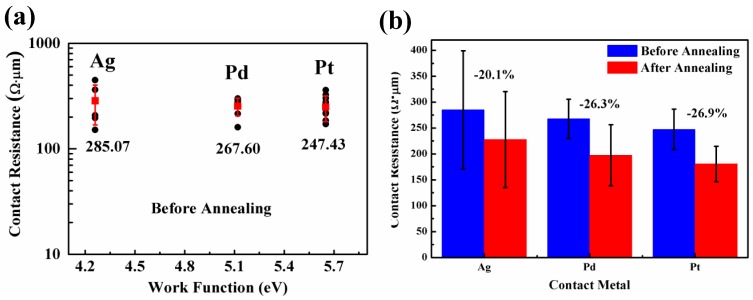
(**a**) Specific contact resistance as a function of metal work functions (Ag, Pd, and Pt). Filled black circles (●) represent extracted R_c_ values for different metals (Ag, Pd, and Pt). Filled red squares (■) depict the mean R_c_ values with error bar; (**b**) Extracted average R_c_ values with error bar for all G-M contacts (Ag, Pd and Pt/graphene) before and after annealing.

Except the plot of R_c_ with different metal work functions [35,36] in Figure 5a for three G-M contacts before annealing, all extracted R_c_ data in Figure 4 are summarized in Figure 5b for the sake of easy comparison. In Figure 5a, filled black circles represent the extracted R_c_ values for different metals (Ag, Pd, and Pt) and filled red squares depict the mean R_c_ values with error bar. As seen, albeit the metal work function alters from ~4.26 to ~5.65 eV for different metals, the R_c_ value remains nearly invariable, manifesting that R_c_ is barely—or even not—relevant to metal work functions before annealing. This observation is also in accordance with previous finds of E. Watanabe’s et al. [37] in which different G-M contacts were investigated. It seems that the work function of the graphene under different metals is pinned to a particular value regardless of the work function of used metals as indicated in [38].

**Figure 6:**
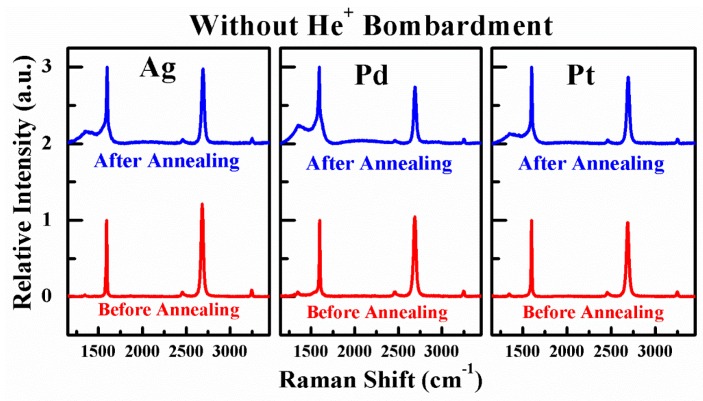
The Raman spectra of the graphene in the G-M contact windows before and after annealing.

By comparing R_c_ values for all G-M contact before and after annealing shown in Figure 5b, it is obvious that thermal annealing is helpful in reducing G-M contact resistance. In [30], it is argued that the mechanism behind improved G-M contact property after thermal annealing is, however, not attributed to the removal of resist residues, instead to numerous end-contacts between metals and dangling bonds in graphene formed by the reaction of dissolved carbon atoms from graphene lattice sites with metals [30]. It is not unexpected for Ni used in [30] that the R_c_ value is reduced significantly after annealing since it is a chemically adsorbed metal. The intriguing point in this work is why physically adsorbed metals like Pt and Ag also show remarkably reduced R_c_ values after annealing, i.e., from 247.43 and 285.07 to 180.75 and 227.81 Ω·μm for Pt/graphene and Ag/graphene contacts, respectively. In order to clarify this point, elaborate characterizations of graphene in G-M contact windows before and after annealing are implemented by Raman scattering spectrum. The contact windows are opened using aqua regia (HCl:HNO_3_ = 3:1) to remove metals thus exposing the graphene under metals [30]. The Raman spectra of as-exposed graphene (before and after annealing) are displayed in Figure 6. As obviously seen, for all G-M contacts (Ag, Pd, and Pt), a distinct D peak appears at ~1350 cm^−1^ for the graphene after annealing in comparison to the graphene before annealing. Note that the Raman spectra of the graphene before annealing resemble those of as-transferred graphene on SiO_2_ (cf. Figure 1c), indicating that the D peak does not originate from the metal deposition and aqua regia attack and this is also in consistent with Ref. [30]. Consequently, the appearance of D peak can be solely attributed to the structural defects in the graphene after annealing. As aforementioned, these structural defects result from the formation of numerous end-contacts between graphene and metals after annealing and this enhances G-M contact property prominently. Along with this guideline, the presence of end-contacts between graphene and metals is critical to achieve extremely low specific contact resistance for G-M contacts. The approach to introduce defects thus forming end-contacts is, therefore, naturally brought up which is investigated in the following part. In the following part, a self-aligned He^+^ bombardment method to induce defects in the graphene within the contact windows and therefore to form end-contacts after annealing is explicitly illustrated.

### 3.2. Effect of He^+^ Bombardment on the G-M Contact Properties

**Figure 7:**
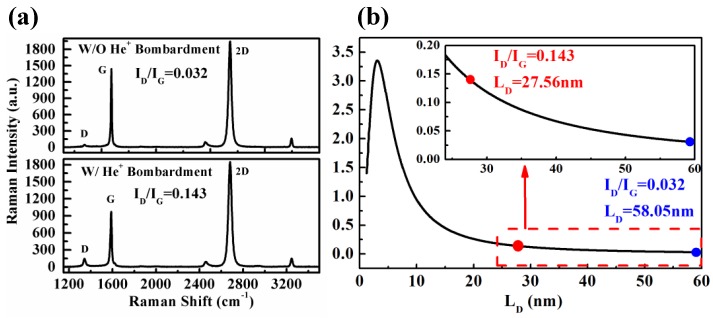
(**a**) Raman spectra of the graphene in G-M contact windows with/without He^+^ bombardment before metal depositions; (**b**) The calculated relationship between I_D_/I_G_ and mean defect distance (L_D_).

The graphene in G-M contact windows was bombarded by He^+^ to intentionally induce defects within it. The energy and dose of impinging He^+^ is 35 keV and 2 × 10^13^ cm^−2^. After He^+^ bombardment, the graphene in the contact windows is inspected by Raman scattering spectrum. The Raman spectra of the bombarded and un-bombarded graphene as reference are shown in Figure 7a. Qualitatively, the D peak for the bombarded graphene is much more pronounced compared to that for the un-bombarded one, illustrating that the graphene is indeed impaired by the He^+^ bombardment. To reckon quantitatively the damage created by He^+^ bombardment in the graphene, a parameter I_D_/I_G_ is given in Equation (2) [39]:

(2) $$\frac{I_{D}}{I_{G}}=C_{A}\frac{r_{A}^{2}-r_{S}^{2}}{r_{A}^{2}-2r_{S}^{2}}\left[\exp\left(-\frac{{\pi r}_{S}^{2}}{L_{D}^{2}}\right)-\exp\left(-\frac{\pi \left(r_{A}^{2}-r_{S}^{2}\right)}{L_{D}^{2}}\right)\right]+C_{S}\left[1-\text{exp}\left(-\frac{{\pi r}_{S}^{2}}{L_{D}^{2}}\right)\right]$$

where r_S_ and r_A_ are length scales that determine the region where the D band scattering takes place. r_S_ determines the radius of the structurally disordered area and r_A_ is the radius of the area surrounding the point defects in which the D band scattering takes place. C_A_ is a measure of the maximum possible value of the I_D_/I_G_ ratio in graphene. C_S_ is the value of the I_D_/I_G_ ratio in the highly disordered limit [39,40]. L_D_ is the mean defect distance and the defect density is proportional to 1/L_D_^2^. In accordance with the empirical data in [39], C_A_ = (4.2 ± 0.1), C_S_ = (0.87 ± 0.05), r_A_ = (3.00 ± 0.03) nm, and r_S_ = (1.00 ± 0.04) nm, the I_D_/I_G_ as a function of L_D_ for the graphene with and without He^+^ bombardment is plotted in Figure 7b. It can be seen that as the I_D_/I_G_ increases from 0.032 for the un-bombarded graphene to 0.143 for the bombarded one, the L_D_ increases dramatically from 58.05 nm to 27.56 nm. This means that the defect density in the graphene is increased approximately 4.4 times using He^+^ bombardment.

**Figure 8:**
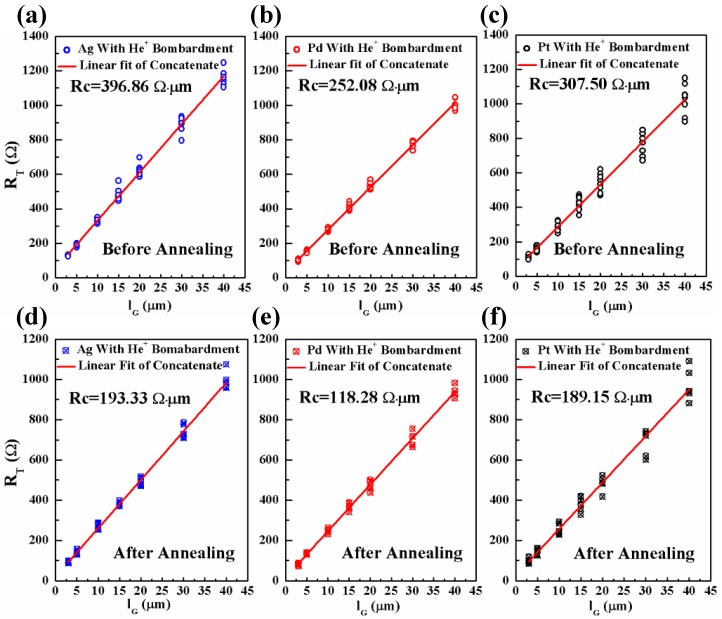
The linear plots of R_T_ vs. l_G_ for the Ag, Pd, and Pt/graphene contacts with He^+^ bombardment before annealing (**a**–**c**) and after annealing (**d**–**f**). For each l_G_, 10 data points are used and the liner fitting is performed using concatenate fit method. Extracted R_c_ value is shown in each corresponding plot.

**Figure 9:**
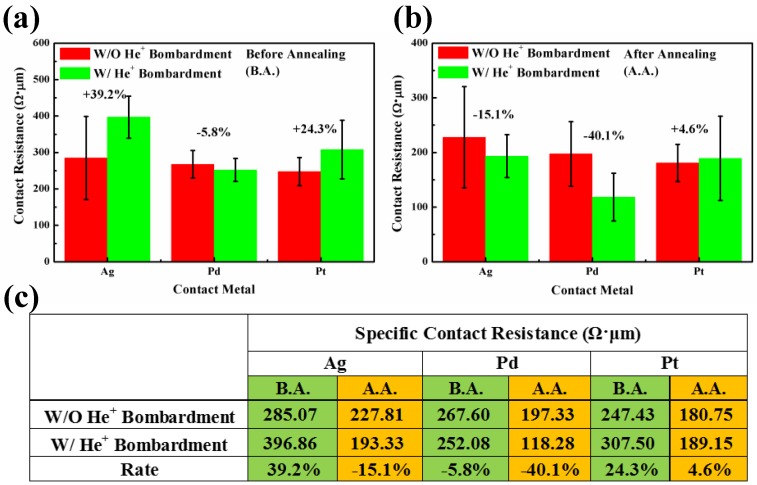
The comparison of R_c_ for three G-M contacts with and without He^+^ bombardment (**a**) before annealing and (**b**) after annealing; (**c**) Detailed R_c_ values in (**a**,**b**) are summarized. B.A.: Before Annealing; A.A.: After Annealing.

For Ag, Pd, and Pt/graphene contacts with He^+^ bombardment, the linear plots of R_T_ vs. l_G_ before annealing (a–c) and after annealing (d–f) are shown in Figure 8. Similarly to the G-M contacts without He^+^ bombardment, 10 data points are used and the linear fitting is performed using concatenate fit method. As seen, the effectiveness of annealing in improving the G-M contacts with He^+^ bombardment is also evident. However, how the He^+^ bombardment impacts the specific contact resistance is still not clear. In order to provide a direct comparison for the readers, the R_c_ values for the G-M contacts with/without He^+^ bombardment are summarized in Figure 9. In Figure 9a, the comparison is made for three G-M contacts with and without He^+^ bombardment before annealing. It is obvious that except Pd, He^+^ bombardment leads to an increase in R_c_ value for the Ag/graphene and Pt/graphene contacts. The defects induced by He^+^ bombardment should be blamed for the increase of R_c_ value and this observation also agrees well with the G-M contacts formed by metal sputtering on graphene [41]. The defects in graphene will probably result in the scattering of carriers and thereof the carriers’ mean free path is shortened, which in turn, gives rise to the increase of R_c_ [11]. In Figure 9b, the comparison is made for three G-M contacts with and without He^+^ bombardment after annealing. As seen, for Ag/graphene and Pd/graphene contacts with He^+^ bombardment, the R_c_ values are reduced dramatically by 15.1% and 40.1% compared to their counterparts without He^+^ bombardment. Note that the obtained R_c_ values in this work for all three G-M contacts, i.e., 193.33, 118.28 and 189.15 Ω·µm for Ag, Pd, Pt/graphene contacts respectively, are among the lowest specific contact resistances [11,29,37]. Nevertheless, for Pt/graphene contact with He^+^ bombardment after annealing, the R_c_ value is increased a little bit compared to its counterpart without He^+^ bombardment and this could be tentatively interpreted as what follows.

**Figure 10:**
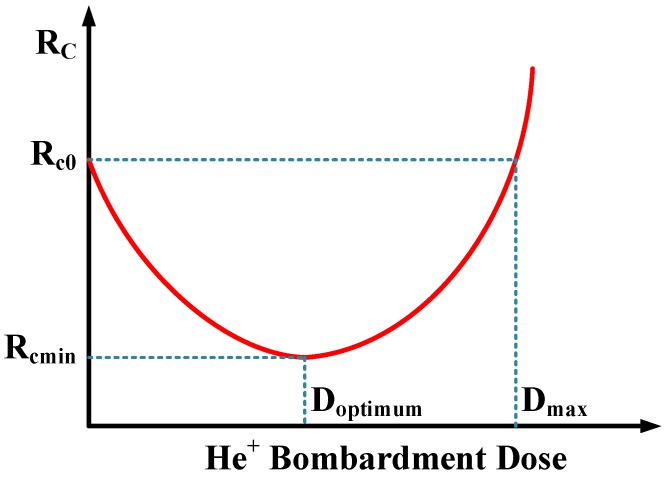
Schematic representation of the specific contact resistance (R_c_) after annealing vs. He^+^ bombardment dose.

In Figure 10, an attempt is made to schematically illustrate why the R_c_ value is lower for Ag/graphene and Pd/graphene contacts whereas is higher for Pt/graphene with He^+^ bombardment after annealing. We think the discussion about the higher or lower of contact resistance after annealing should take both the capability of metals forming end-contacts with defects and carrier scattering induced by defects within graphene into account [41,42,43,44]. It is assumed that for each G-M contact there is an optimum dose (D_optimum_) of He^+^ bombardment where the lowest R_c_ value (R_cmin_) takes place. Four cases happen to the dose of He^+^ bombardment. (1) If He^+^ dose is D_optimum_, the created defects in graphene can be completely consumed by the formation of end-contacts between metals and graphene. As a result, R_cmin_ can be obtained; (2) If He^+^ dose is less than D_optimum_, R_c_ value is reduced but not to R_cmin_ in that as-formed end-contacts are not saturated; (3) On the other hand, if He^+^ dose is more than D_optimum_ but less than D_max_, though the formation of saturated end-contacts leads to the reduction of R_c_, excess created defects enhance the carriers scattering which unfortunately degrades R_c_ concurrently. The joint efforts of as-formed end-contacts and excess created defects render the R_c_ value not the lowest R_cmin_; (4) If He^+^ dose is more than D_max_, the effort of excess created defects prevails over that of saturated end-contacts and this leads to drastically increased R_c_ value that is even larger than R_c0_ i.e., the R_c_ value for G-M contacts without He^+^ bombardment after annealing. Specifically, for the Ag/graphene and Pd/graphene contacts, they belong to cases (2) and (3) where their R_c_ values are reduced but possibly not to the lowest values R_cmin_. It is worth noting that for Pd/graphene contact, the employed dose 2 × 10^13^ cm^−2^ is even closer to its D_optimum_ since its reduction percentage 40.1% is much bigger than 15.1% for Ag/graphene contact. For Pt/graphene contact, it belongs to case (4) where the effort of excess created defects prevails over that of as-formed saturated end-contacts, resulting in even larger R_c_ value compared to its counterpart without He^+^ bombardment after annealing. The specific contact resistance as a function of ion bombardment dose is still under investigation and will be revealed as a continuation of this work in the near future.

**Figure 11:**
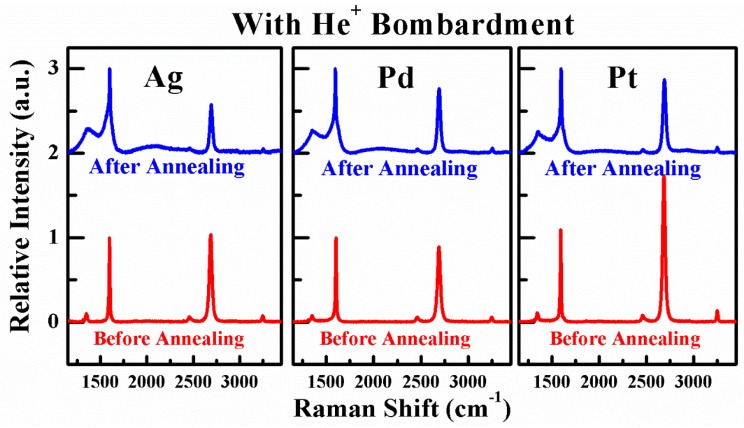
The Raman spectra of the graphene in the G-M contact windows with He^+^ bombardment before and after annealing.

Similarly to the discussions in previous section, the elaborate characterizations of graphene in G-M contact windows with He^+^ bombardment before and after annealing are also performed by Raman scattering spectrum. The Raman spectra of as-exposed graphene with He^+^ bombardment (before and after annealing) are depicted in Figure 11. As can be seen, for all Ag, Pd, and Pt/graphene contacts, a tiny D peak appears at ~1350 cm^−1^ for the graphene before annealing in comparison to as-transferred graphene in Figure 1c and this is ought to be the result of He^+^ bombardment. However, for the graphene with He^+^ bombardment after annealing, the D peak becomes startlingly conspicuous. If scrutinized, the intensities of D peak for the graphene with He^+^ bombardment in all G-M contact windows are higher than those for the graphene without He^+^ bombardment in Figure 6. This observation is good evidence that the formation of end-contacts is favorable in the presence of He^+^ bombardment. We believe the key to achieve extremely low specific contact resistance is to introduce defects in graphene thus forming plenty of end-contacts after annealing like other methods to form end-contacts [20,27,28]. The approaches to introduce defects into graphene can be diverse, not only by He^+^ bombardment, but also by the implantation of other ion species. It is a remarkable fact that the optimum dose to achieve the lowest specific contact resistance may vary depending on the ion species used or on the implantation energy.

## 4. Conclusions

To summarize, the specific contact resistance for three G-M contacts (Ag, Pd, and Pt/graphene) are all reduced after annealing from 285.07, 267.60, and 247.43 Ω·µm to 227.81, 197.33, and 180.75 Ω·µm, respectively. This indicates that not only chemically adsorbed metal (Pd) but also physically adsorbed metals (Ag and Pt) readily form end-contacts at intrinsic defect locations in graphene. Along with this guideline, a novel method, in which self-aligned He^+^ bombardment to induce exotic defects in graphene and subsequent thermal annealing to form end-contacts, was proposed in order to further reduce the specific contact resistance. Achieved results show that the specific contact resistances are reduced significantly by 15.1% and 40.1% for Ag/graphene and Pd/graphene contacts with He^+^ bombardment compared to their counterparts without He^+^ bombardment, respectively. For the Pt/graphene contact, the contact resistance is, however, not reduced as anticipated with He^+^ bombardment and this might be ascribed to either inappropriate He^+^ bombardment dose, or inapplicable method of He^+^ bombardment in reducing contact resistance for Pt/graphene contact. The effort of as-formed end-contacts prevailing over that of excess created defects is attributed to the reduction in R_c_ values for G-M contacts with He^+^ bombardment after annealing. By manipulating the He^+^ bombardment dose in conjunction with Ag, Pd, and Pt metals, the lowest R_c_ value for each G-M contact could be possibly accomplished. It is worth noting that the proposed He^+^ bombardment and metal depositions share the same lithography mask and this processing simplicity demonstrates that our proposed method is very efficient in improving the contact properties for graphene-based devices in the future.
